# Elevated Serum Soluble Syndecan-1 Is Associated with Lupus Nephritis Flares: A Cross-Sectional Study

**DOI:** 10.3390/ijms27093851

**Published:** 2026-04-26

**Authors:** Nicte Selene Fajardo-Robledo, Heriberto Jacobo-Cuevas, Juan Manuel Ponce-Guarneros, Soraya Amalí Zavaleta-Muñiz, Erika Anita Aguilar-Chavez, Alberto Daniel Rocha-Muñoz, Edy David Rubio-Arellano, Juan Manuel Viveros-Paredes, Eva Maria Olivas-Flores, Felipe Alexis Avalos-Salgado, Aniel Jessica Leticia Brambila-Tapia, Fabiola Gonzalez-Ponce, Ernesto German Cardona-Muñoz, Laura Gonzalez-Lopez, Jorge Ivan Gamez-Nava

**Affiliations:** 1 Laboratorio de Investigación y Desarrollo Farmacéutico, Departamento de Farmacobiología, Centro Universitario de Ciencias Exactas e Ingenierías, Universidad de Guadalajara, Guadalajara 44430, Jalisco, Mexico; nicte.fajardo@academicos.udg.mx (N.S.F.-R.); juan.viveros@academicos.udg.mx (J.M.V.-P.); alexis.avalos@academicos.udg.mx (F.A.A.-S.); 2 Cuerpo Académico UDG-CA-1061 “Farmacología Traslacional”, Centro Universitario de Ciencias Exactas e Ingenierías, Universidad de Guadalajara, Guadalajara 44430, Jalisco, Mexico; 3 Group for the Assessment of Prognosis Biomarkers in Autoimmune Disorders, Centro Universitario de Ciencias de la Salud, Guadalajara 44340, Jalisco, Mexico; hjacobocuevas@gmail.com (H.J.-C.); juan.ponce4091@academicos.udg.mx (J.M.P.-G.); medalbertorocha@hotmail.com (A.D.R.-M.); eolivasflores@gmail.com (E.M.O.-F.); fabiola.gonzalez@academicos.udg.mx (F.G.-P.); cameg1@gmail.com (E.G.C.-M.); 4 Programa de Postdoctorado en el Departamento de Psicología Básica, Centro Universitario de Ciencias de la Salud, Universidad de Guadalajara, Guadalajara 44340, Jalisco, Mexico; 5 Programa de Doctorado en Farmacología, Departamento de Fisiología, Instituto de Terapéutica Experimental y Clínica, Centro Universitario de Ciencias de la Salud, Universidad de Guadalajara, Guadalajara 44340, Jalisco, Mexico; edavidrubio@gmail.com; 6 Cuerpo Académico UDG-CA-1172 “Alteraciones Inmunologicas y Enfermedades Cronico-Degenerativas”, Centro Universitario de Ciencias de la Salud, Guadalajara 44340, Jalisco, Mexico; 7 Facultad de Ciencias de la Salud, Universidad Juárez del Estado de Durango, Gómez Palacio 35050, Durango, Mexico; soraya.zavaleta@ujed.mx; 8 Unidad de Medicina Familiar 2, Instituto Mexicano del Seguro Social, Guadalajara 44340, Jalisco, Mexico; anita.aguilar@academicos.udg.mx; 9 Centro Universitario de Tonalá, Universidad de Guadalajara, Tonalá 45425, Jalisco, Mexico; 10 Departamento de Anestesiología, Hospital de Especialidades, Centro Medico Nacional de Occidente, IMSS, Guadalajara 44340, Jalisco, Mexico; 11 Departamento de Psicología Básica, Centro Universitario de Ciencias de la Salud, Universidad de Guadalajara, Guadalajara 44340, Jalisco, Mexico; aniel.brambila@academicos.udg.mx

**Keywords:** syndecan-1, anti-dsDNA, lupus nephritis, proteinuria, sensitivity, specificity

## Abstract

Lupus nephritis (LN) is a severe immune-mediated renal disorder causing significant morbidity and mortality in patients with systemic lupus erythematosus (SLE). Traditional biomarkers (serum complement components C3 and C4 and anti-dsDNA antibodies) have limited sensitivity and specificity for detecting renal flares; thus, new markers are needed to improve relapse detection and therapeutic response monitoring. We conducted a cross-sectional study including 71 women with SLE and 20 age- and sex-matched controls. Clinical data were collected, global activity was evaluated using the SLEDAI, and renal activity was evaluated using the renal SLEDAI (rSLEDAI). Serum syndecan-1 (SDC-1) and anti-dsDNA were measured using ELISA, and 24 h proteinuria was quantified. According to the rSLEDAI, 38 patients (53.5%) had LN flare, with a mean SDC-1 level of 108.5 ± 69.3 ng/mL and anti-dsDNA level of 113.1 ± 148.8 IU/mL. SDC-1 was correlated with the rSLEDAI (r = 0.32; *p* = 0.006), prednisone dose (r = 0.37; *p* = 0.002), proteinuria (r = 0.33; *p* = 0.005), and anti-dsDNA (r = 0.33; *p* = 0.006), while anti-dsDNA was positively correlated with proteinuria (r = 0.39; *p* = 0.001 and SDC-1 (r = 0.33; *p* = 0.006) and negatively correlated with age (r = −0.33; *p* = 0.006). High SDC-1 (cutoff ≥ 89 ng/mL) had higher sensitivity for detecting renal flares than anti-dsDNA (66% vs. 45%). In the multivariable analysis, high SDC-1 levels had around a 3-fold higher risk of being associated with LN flares, independently of anti-dsDNA and complement component levels. These results support serum SDC-1 as a promising biomarker for identifying renal flares in SLE patients, and it should be combined with traditional biomarkers to increase its value as a clinical tool. Follow-up studies are required to determine its value for predicting long-term renal outcomes.

## 1. Introduction

Systemic lupus erythematosus (SLE) is an autoimmune disease characterized by a loss of tolerance to numerous autoantigens, leading to systemic inflammation and immune-mediated injury in multiple organs, particularly the kidneys [1,2]. Lupus nephritis (LN) is one of the most common and severe manifestations of SLE and results in significant morbidity and mortality. Between 50 and 60% of patients with SLE develop nephropathy, with around 15% of cases progressing to end-stage renal disease (ESRD) [3]. LN can also cause mortality, with a 10-year survival rate between 81 and 98% [4]. Flares of active nephritis are the strongest drivers of the progression to kidney failure requiring hemodialysis or renal transplant [1].

In clinical practice, an early detection of renal flares is important to modify treatments, improving the rate of response and avoiding the impairment in renal function. Traditionally, clinicians have used serum biomarkers, such as low serum complement component levels (C3 and C4) and anti-dsDNA antibodies; however, they have several limitations, including limitations in sensitivity and specificity for detecting LN flares in the early stages of development in some patients. Overall, the reported utility values for low C3 vary across the studies with a sensitivity that goes from 70% to 100% and specificity that goes from 50.9% to 71%; whereas for low C4, the sensitivity can vary from 48% to 100% and specificity from 62.3% to 71%. The most useful traditional test for the clinicians seems to be the anti-dsDNA; however, the performance of this test varies depending on the platform used (*Crithidia luciliae* indirect immunofluorescence test, Farr radioimmunoassay, Chemiluminescence Immunoassay, Fluorescent Enzyme Immunoassay, or Enzyme-Linked Immunosorbent Assay (ELISA)); of them, the most used by practicality is the determination of anti-dsDNA by ELISA. Using this method the sensitivity of anti-dsDNA for renal flares goes from 40% to 91.9%, whereas the specificity varies from 36.5% to 90%. Consequently, the Positive Predictive Values (PPVs)—defined as the probability that a person with SLE truly has a renal flare when the serum marker test result is positive—reported for low C3 (22–23%), low C4 (17–28%), and anti-dsDNA (42–80%) in identifying renal flares are relatively low [5,6,7]. Furthermore, these biomarkers are limited in distinguishing active disease from chronic organ damage, which is crucial for treatment planning [8].

Therefore, new serum biomarkers with better performance in sensitivity and PPV would improve the ability to detect relapses, thus supporting early diagnosis and subsequent therapeutic response.

Certain components of the endothelial glycocalyx have been found to play a role in vascular damage and systemic inflammation in several nephropathies, including idiopathic nephrotic syndrome, proteinuria with minimal change disease, and LN [9,10]. One such component is syndecan-1 (SDC-1), also known as CD-138, a transmembrane heparan and chondroitin sulfate proteoglycan that is a major structural component of the endothelial and epithelial glycocalyx. Through its glycosaminoglycan (GAG) side chains, it mediates interactions with growth factors, chemokines, and adhesion molecules, thereby preserving the structural integrity of the glomerular filtration barrier and maintaining endothelial homeostasis [11].

During active LN, pro-inflammatory cytokines such as TNF-α and IL-6, together with matrix metalloproteinases (MMPs), induce ectodomain shedding of SDC-1, leading to its soluble form. This change is a hallmark of endothelial glycocalyx degradation. The shedding process increases vascular permeability, facilitates leukocyte infiltration, and amplifies the inflammatory response through the release of growth factors and cytokines previously sequestered within heparan sulfate chains [12].

In LN, it has been observed that the increase in plasma SDC-1 is higher in SLE cases. Studies in patients with SLE have shown that serum levels of SDC-1 are higher in a state of active LN than in non-renal SLE, LN in remission, non-lupus chronic kidney disease, and healthy controls, with significant correlations with proteinuria, anti-dsDNA, complement component C3, and renal function [13].

The aim of this study was to determine whether serum SDC-1 is a potential biomarker for LN and to identify the value of serum SDC-1 as a risk factor of renal flares in comparison to traditional biomarkers such as positive anti-dsDNA or low complement components C3 and C4 by adjusting for cofounders in a multivariable approach.

## 2. Results

### 2.1. General Characteristics of SLE Patients

Seventy-one patients were evaluated, all of whom were female. The group had a mean age of 43.0 ± 10.6 years and a mean disease duration of 7.9 ± 5.1 years. In Table 1 are shown the clinical characteristics of SLE when this study was conducted. At the time of this study, 38 patients had developed a renal relapse manifested by proteinuria >0.5 g/24-h in 39.4%, leukocyturia >5 white blood cells/HPF in 23.9%, hematuria >5 red blood cells/HPF in 4.2%, urinary casts in 1.4%, and elevated serum creatinine ≥ 1.5 (mg/dL) in 7.0%. Regarding laboratory findings, 33.8% of patients had positive anti-dsDNA antibodies (Table 1).

According to the renal SLEDAI, 38 patients (53.5%) had renal disease activity. The mean SDC-1 level was 108.5 ± 69.3 ng/mL, and the mean anti-dsDNA count was 113.1 ± 148.8 UI/mL (Table 1).

### 2.2. Comparison of SDC-1 Levels Between LN, Inactive SLE, and Healthy Controls (HC)

**Figure 1:**
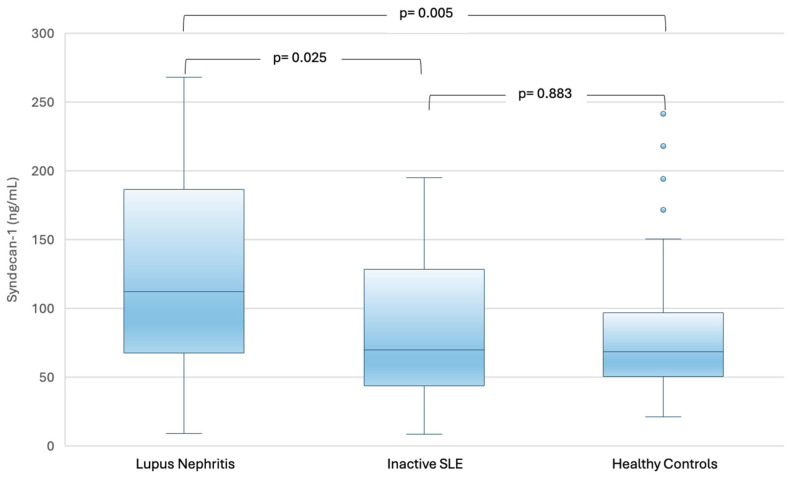
A comparison of serum syndecan-1 levels in patients with lupus nephritis, patients with inactive SLE, and healthy controls. Differences between two groups (i.e., lupus nephritis vs. inactive SLE) were analyzed using the Mann–Whitney U test. Serum SDC-1 levels were significantly higher in patients with lupus nephritis than in patients with inactive SLE (*p* = 0.025) and healthy controls (*p* = 0.005), whereas no significant differences were observed between patients with inactive SLE and healthy controls (*p* = 0.883).

In Figure 1, a comparison of serum SDC-1 levels between the group of LN, inactive SLE and healthy controls (HC) is shown. As it was observed in the figure, the group of LN had higher concentrations of serum SDC-1 compared to the other groups. There were no differences in serum SDC-1 between inactive SLE patients and HC.

### 2.3. Correlations of Serum SDC-1 and Anti-dsDNA with Selected Variables

Table 2 shows the correlations of serum SDC-1 levels and anti-dsDNA with clinical variables. SDC-1 levels were correlated with the rSLEDAI score (r = 0.32; *p* = 0.006); daily dose of prednisone (r = 0.37; *p* = 0.002); urinary proteins, expressed as g/24-h (r = 0.33; *p* = 0.005); and anti-dsDNA levels (r = 0.33; *p* = 0.006). Anti-dsDNA levels were positively correlated with urinary proteins, expressed as g/24 h (r = 0.39; *p* = 0.001), and SDC-1 levels (r= 0.33; *p*= 0.006) and negatively correlated with age (r = −0.33; *p* = 0.006).

### 2.4. Receiver Operating Characteristic (ROC) Curve Analysis of Serum Biomarkers

**Figure 2:**
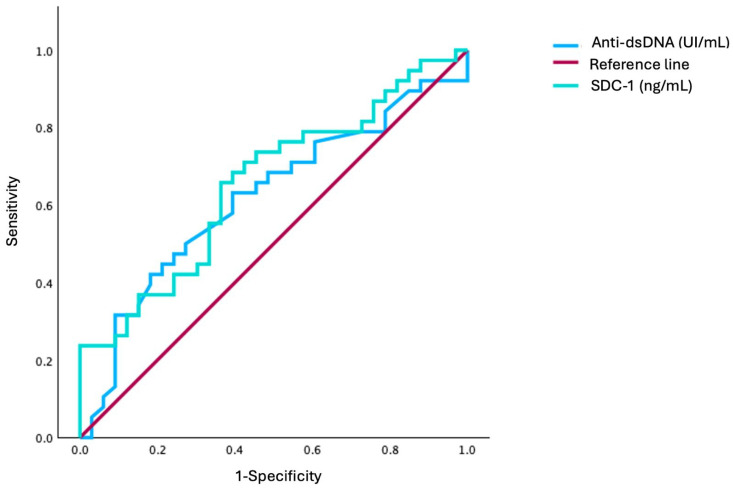
ROC curve analysis comparing SDC-1 and anti-dsDNA in identifying lupus nephritis in SLE patients. The ROC curve for serum SDC-1 in lupus nephritis patients has an area under the curve of 0.66 (CI 95%: 0.53–0.78), with a serum SDC-1 cutoff of ≥ 89.0 ng/mL meaning high levels, and that for anti-dsDNA has an area under the curve of 0.62 (CI 95%: 0.49–0.75), with a cutoff for positive anti-dsDNA of ≥100 UI/mL.

In Figure 2, it is observed that the ROC curve analysis of serum SDC-1 and anti-dsDNA shows the discrimination between LN (n = 38) and inactive SLE (n = 33). The ROC curve for serum SDC-1 in LN had an area under the curve (AUC) of 0.66, and anti-dsDNA had an AUC of 0.62. The Youden’s index identified a cutoff for SDC-1 of ≥ 89.0 ng/mL, whereas, for positive anti-dsDNA, it was ≥100 UI/mL.

### 2.5. Utility Values as a Diagnostic Test of SDC-1 and Anti-dsDNA

Table 3 outlines the utility of SDC-1 and anti-dsDNA for identifying LN flares. The highest sensitivity for identifying LN (66%) was observed for SDC-1 (cutoff ≥ 89 ng/mL); whereas the highest specificity (79%) was observed for anti-dsDNA (cutoff ≥ 100 UI/mL). The PPV value was also higher with the anti-dsDNA ≥100 UI/mL (71%), whereas the highest negative predicted value (NPV) was for SDC-1. The positive Likelihood Ratio (LR+) indicates how much the odds of disease increase when a test of SDC-1 or anti-dsDNA is positive. Both SDC-1 (LR+: 1.81) and anti-dsDNA (LR+: 2.11) indicate a small change in likelihood of having renal flares, whereas the negative Likelihood Ratio (LR−) for SDC-1 (LR−: 0.54) and for anti-dsDNA (LR−: 0.70) indicates a limited contribution for excluding renal flares in patients with levels under the cutoff of these markers.

### 2.6. Comparison of Selected Variables Between LN and Inactive SLE

Table 4 shows a comparison between the clinical and laboratory variables in LN and inactive SLE. No significant differences were observed in age, disease duration, or BMI between groups (*p* > 0.05). As expected, disease activity measured using the SLEDAI was significantly higher in LN patients than in inactive SLE patients (7.0 ± 3.0 vs. 0.5 ± 0.8, *p* < 0.001). A higher proportion of LN patients received GC doses >10 mg/day than inactive SLE patients (73.7% vs. 45.5%, *p* = 0.016). The use of immunosuppressive drugs did not significantly differ between groups.

Serum markers of disease activity such as positive anti-dsDNA ≥ 100 IU/mL were more prevalent in the LN group than in the inactive SLE group (44.7% vs. 21.2%, *p* = 0.037). C4 complement component levels were significantly lower in the LN group than in the inactive SLE group (21.0 ± 6.8 vs. 25.9 ± 10.2 mg/dL, *p* = 0.018), whereas there were no differences in C3 complement component levels between the groups. Finally, serum SDC-1 levels were significantly higher in the LN group than in the inactive SLE group (127.4 ± 76.2 vs. 86.7 ± 53.5 ng/mL, *p* = 0.011).

### 2.7. Multivariable Analysis of Factors Associated with Flares of LN

Table 5 shows the results of a multivariable logistic regression analysis, where the dependent variable was the presence of an LN flare (rSLEDAI ≥ 4). In this logistic regression, high levels of serum SDC-1 (≥89 ng/mL), positive anti-dsDNA ≥ 100 UI/mL, and low levels of the serum complement component C3 or C4 were introduced as risk factors for LN, with adjustment for age and SLE disease duration. Using the enter method, it was found that serum SDC-1 levels ≥ 89 ng/mL were significantly associated with LN (OR = 2.91, 95% CI: 1.04–8.17, *p* = 0.04). Although anti-dsDNA levels ≥100 IU/mL showed a trend toward association (OR = 2.66, 95% CI: 0.81–8.71), this did not reach statistical significance (*p* = 0.10). Instead, complement components C3 and C4 were not associated with LN in this analysis. Finally, using the forward stepwise method, it was found that high levels of SDC-1 remained a major independent predictor of LN (OR = 3.37, 95% CI: 1.27–8.93, *p* = 0.02). All other variables were excluded from the final model due to a lack of statistical significance.

## 3. Discussion

In this study, we observed that serum SDC-1 levels were higher in patients with LN than in patients with inactive SLE and healthy controls. Furthermore, SDC-1 levels were correlated with proteinuria and anti-dsDNA titers. When examining the utility of high levels of serum SDC-1 (≥89 ng/mL), the positive predictive value was found to be 68%, with a sensitivity of 66% for detecting LN and a specificity of 64% for excluding this complication. Finally, after adjusting for age, disease duration, and other potential confounders, it was found that high serum SDC-1 levels (≥89 ng/mL) were associated with an approximately 3-fold higher risk of LN. Therefore, the results of this study suggest that SDC-1 levels are a useful marker for LN complementary to other traditional serum markers, such as positive anti-dsDNA and low levels of C3 and C4 complement components.

SDC-1 (CD138) is a transmembrane heparan sulfate proteoglycan widely expressed on the cell surface and, under specific conditions, localized to the nuclear membrane [14]. It interacts with a variety of ligands such as growth factors, cytokines, proteinases, adhesion molecules, and extracellular matrix (ECM) components [12].

SDC-1 represents the most extensively characterized member of the four-member syndecan family of heparan sulfate proteoglycans. In chronic inflammation, it modulates the immune response through several mechanisms: (a) it interacts with chemokines and adhesion molecules, such as E-selectin, leading to leukocyte extravasation and migration to sites of inflammation, thereby favoring neutrophil and monocyte recruitment with subsequent tissular damage, and (b) it exerts an anti-inflammatory effect through ectodomain shedding, releasing soluble SDC-1 molecules [12].

**Figure 3:**
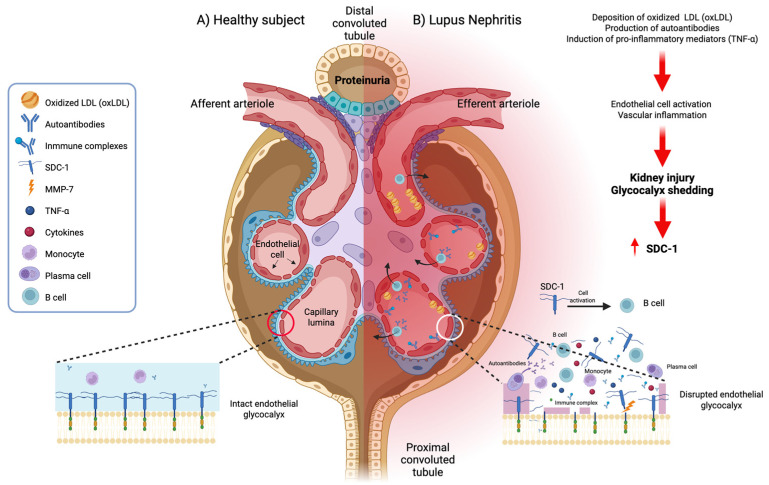
Hypothetical mechanism of syndecan-1 in lupus nephritis in systemic lupus erythematosus (SLE). (**A**) Normal function and (**B**) lupus nephritis—the combined effects of the accumulation of oxidized low-density lipoprotein (OxLDL), the production of autoantibodies (e.g., anti-dsDNA), and the induction of pro-inflammatory mediators such as TNF-α trigger endothelial cell activation, immune complex deposition, and vascular inflammation, leading to renal damage and glycocalyx shedding, thereby increasing soluble SDC-1 levels, which, in turn, perpetuate inflammation through the activation of immune cells such as B cells. Figure created in BioRender.com. Jacobo-Cuevas H, (2026) https://app.biorender.com/illustrations/69d840d4d93c5016d6b55fba?slideId=5ab9dde9-d2a0-4da8-9923-33196dff4140.

Soluble SDC-1 contributes to tissue injury through multiple interconnected mechanisms, as illustrated in Figure 3, affecting the glomerular glycocalyx, autoantibody production, the inflammatory milieu, tubular epithelial integrity, and growth factor signaling [15,16]. Soluble SDC-1 is a key structural element of the endothelial glycocalyx, and, in LN, systemic inflammation increases its shedding, resulting in degradation of this protective layer and the exposure of adhesion molecules such as VCAM-1 and ICAM-1 on the luminal surface of glomerular endothelial cells, thereby facilitating leukocyte adhesion and migration into the kidney [3,15]. Circulating soluble SDC-1 also functions as a potent B-cell activator, promoting B-cell differentiation and, in concert with other mediators, driving the generation of pathogenic autoantibodies, such as anti-dsDNA, which contribute to the onset and exacerbation of SLE and LN flares [17,18,19]. In addition, the heparan sulfate chains released on soluble SDC-1 stimulate the production of pro-inflammatory cytokines and low-molecular-weight hyaluronic acid, intensifying inflammatory responses [15,20]. In the kidney, the shedding of SDC-1 compromises adherent junctions between tubular epithelial cells, largely through increased STAT3 activation; meanwhile, loss of membrane-bound SDC-1 decreases E-cadherin expression, causing a loss of cell polarity, increased apoptosis, and progression toward fibrosis [21]. Finally, soluble SDC-1 can act as a sink for growth factors such as VEGF in the circulation, and, by sequestering or altering the availability of these factors, it interferes with normal tissue repair mechanisms and promotes vascular dysfunction and fibrotic remodeling [15,22].

Additionally, an increase in soluble SDC-1 has been associated with several types of nephritis, such as diabetic nephropathy [23], idiopathic nephrotic syndrome [9], acute kidney injury [24], and LN [13,17,25].

In SLE, the systemic inflammation promotes the cleavage of SDC-1 and its subsequent release into the circulation; this phenomenon is observed in LN, releasing a series of events related to renal inflammation. Kim et al. proposed that residual SDC-1 molecules may activate quiescent autoreactive plasma cells, inducing autoantibody production and potentially contributing to the development of renal flares [3,17].

Soluble SDC-1 contributes to tissue injury through multiple interconnected mechanisms, affecting the glomerular glycocalyx, autoantibody production, the inflammatory milieu, tubular epithelial integrity, and growth factor signaling (Figure 3) [15,16].

In this study, we observed that serum SDC-1 levels were higher in LN patients than in inactive SLE patients and healthy controls. Furthermore, SDC-1 levels were correlated with proteinuria, hematuria, anti-dsDNA levels, and the rSLEDAI score, reflecting the potential role of soluble SDC-1 levels as a biomarker for active renal involvement. Using a cutoff of a high SDC-1 level of 89 ng/mL, we observed a sensitivity of 66% for detecting LN and a specificity of 64% for excluding inactive SLE. After adjusting for age, disease duration, and other potential confounders, it was found that serum SDC-1 levels ≥ 89 ng/mL had a 3-fold higher risk of being associated with LN. Therefore, this study suggests that SDC-1 levels can serve as a good marker for LN.

Several studies have evaluated the relevance of serum SDC-1 levels in SLE. Minowa et al. investigated a cohort of 22 SLE patients, identifying higher serum SDC-1 levels in SLE patients than in controls [18]. These authors found a correlation between SDC-1 levels (CD-138) and the proportion of CD20^−^, CD38^+^, and CD138^+^ plasma cells [18]. In a cross-sectional study, Kim et al. compared serum SDC-1 levels among SLE patients, individuals with rheumatoid arthritis (RA), and controls, finding that they were almost 2-fold higher in SLE patients than in the other groups [17]. Similarly, Salam et al. compared serum SDC-1 levels in 60 SLE patients (20 with nephritis, 20 with other disease activity, and 20 with inactive SLE) against 20 controls, observing that they were higher in the three groups of SLE patients than in the controls. They also noted that the LN subgroup showed the highest levels of this soluble molecule [26]. In our study, we also compared serum SDC-1 levels among three groups: (a) SLE patients with nephritis, (b) SLE patients without disease activity, and (c) controls. Our findings regarding the high levels of this soluble molecule in SLE coincide with those of previous reports observing that, in SLE patients with nephritis, the median SDC-1 levels were almost 2-fold greater than those in SLE patients with inactive disease or controls. Finally, Carnazzo et al. quantified the serum SDC-1 levels in 60 SLE patients, 60 RA patients, and 40 controls, observing higher levels in the SLE and RA groups than in the controls. However, no differences in the levels of this soluble glycoprotein were found between patients with SLE and RA [27]. Our results differ from those obtained by Salam et al. because we did not observe any differences in SDC-1 levels between inactive SLE patients and controls. In contrast, Randa F Salam et al. found a significant increase in SDC-1 levels in SLE patients in remission versus controls [26]. As the results show (Table 4), most patients in remission still take some type of immunosuppressive drug, such as azathioprine, mycophenolate, or cyclophosphamide. We believe that patients with SLE in remission who are receiving immunosuppressive therapy can achieve a therapeutic response that can lead to a decrease in the levels of several pro-inflammatory molecules (including soluble SDC-1), achieving normal values, as observed in our study.

Regarding the studies assessing the relationship between serum SDC-1 levels and LN, most show a correlation between the clinical parameters of nephritis and this molecule. Ebrahim et al. studied 30 SLE patients and 20 controls and identified higher levels of SDC-1 in SLE patients [25]. They also observed correlations between serum SDC-1 and 24 h proteinuria, anti-dsDNA titers, serum creatinine, low levels of the C3 complement component, and the SLEDAI [25]. Salam et al. identified a correlation between high levels of SDC-1 and 24 h proteinuria, low levels of C3 and C4 complement components, and the SLEDAI [26]. Yu et al. conducted a sophisticated study to measure serum levels of SDC-1, hyaluronan, and thrombomodulin in patients with SLE, comparing patients with active LN, patients with LN in remission, patients with non-renal SLE but other extrarenal disease activity, patients with SLE with non-renal involvement in remission, patients with other causes of chronic kidney disease, and healthy subjects. They found that SDC-1 levels were correlated with proteinuria, serum creatinine, anti-dsDNA antibodies, SLEDAI-2K, renal SLEDAI-2K, and low levels of the C3 complement component and serum albumin [13].

Consistent with the above-mentioned studies, we observed correlations between 24 h proteinuria, the renal SLEDAI, and anti-dsDNA. We did not observe correlations between SDC-1 levels and C3 or C4 complement components. Instead, we identified correlations between SDC-1 levels and hematuria, which is a parameter of active sediment in urine analysis, and other clinical parameters, such as age, BMI, corticosteroid dose (mg/day), low platelet count, and high total cholesterol levels, which have not been previously described.

Only a limited number of studies have explored the cutoff for high levels of serum SDC-1. Yu et al., in determining a cutoff, observed that high SDC-1 levels had a sensitivity of 85.19% and a specificity of 86.21% for identifying active LN versus SLE in remission [13]. In our study, using a cutoff of 89 ng/mL, we identified a sensitivity of 66% and a specificity of 64% for the diagnosis of active LN versus lupus in remission. We also identified a positive predictive value of 68%, meaning that the presence of high serum SDC-1 levels identified 68% of patients with active LN. Traditional serum biomarkers used as clinical tools for the diagnosis of LN flares include anti-dsDNA and complement components: C3 and C4 [7]. However, these biomarkers have limited sensitivity and specificity. In the present study, high titers of anti-dsDNA had the following utility values: a sensitivity of 45% and a specificity of 79%. This indicates that many patients with LN flares do not show increased levels of this biomarker. Therefore, new biomarkers are required to complement the performance of traditional ones. As a biomarker for active LN, SDC-1 seems to exhibit similar performance to anti-dsDNA. However, the titers of anti-dsDNA and SDC-1 have a low correlation (0.325, *p* = 0.006), meaning that their tests should be used as complementarily tools for the better identification of renal flares in SLE.

Our study is one of the few to analyze the risk of high titers of serum SDC-1 in LN flares by using a multivariate approach and adjusting for potentially confounding variables. After adjusting for age, disease duration, and low C3 or C4 levels in the multivariable logistic regression, high SDC-1 and anti-dsDNA levels were found to be predictors of active LN. Both biomarkers constitute risk factors for renal flares by around 2.73-fold (anti-dsDNA) and 2.87-fold (high SDC-1), suggesting that SDC-1 could be useful in clinical decision-making.

Our results can be explained if we consider that soluble SDC-1 is the result of shedding from mature plasma cells. This occurs in response to not only specific development and homeostatic signals but also to wound healing and pathophysiological signals, such as inflammatory cytokines and chemokines [27,28], a role that has been well described during LES disease activity, when cytokines are dysregulated and inflammation is persistent.

### 3.1. Strengths

This study found that serum SDC-1 levels may be a useful biomarker for identifying SLE patients at risk of nephritis. Only a few studies have assessed the cutoff of serum SDC-1 levels to identify the utility values of high SDC-1 as a potential biomarker for LN. In a cohort in 31 patients, Yu et al. investigated the relationship between this molecule and biopsy-proven glomerulonephritis III/IV ± V in SLE. They observed that high SDC-1 levels had a sensitivity of 85.19% and a specificity of 86.21% for distinguishing active LN from LN in remission [13]. Our results support these findings, showing that SDC-1 had a higher sensitivity than anti-dsDNA for identifying patients with LN flares. However, in the study conducted by Yu et al., no statistical multivariable approach was used to determine the weight of each biomarker adjusted by potential confounders; then, in our study, in the multivariable analysis, we obtained that high SDC-1 was associated with a 3.36-fold risk of LN flares, whereas neither positive anti-dsDNA nor low complement components C3 and C4 levels reached statistical significance as risk factors for renal flare in this study. In summary, this study provides evidence supporting the use of serum SDC-1 as a complementary clinical biomarker that can be used together with anti-dsDNA or complement components C3 and C4 in patients with suspected renal flares in SLE.

### 3.2. Weaknesses

Despite its strengths, our study has several limitations. First, it was a cross-sectional study, and, as a result, we were unable to identify the relationship between high serum SDC-1 levels and long-term outcomes, such as progressive deterioration of renal function or future development of chronic renal failure. Longitudinal studies are required to assess these potential outcomes. Another limitation is that we included only female patients because this group is preferentially referred to our center. Future studies should include males to verify our findings in that population. Another potential limitation is that we did not perform repeated renal biopsy at the time of assessment. Therefore, we were unable to relate the serum SCD-1 levels to the histological patterns observed in renal biopsy. However, the first aim of this study was to determine whether serum SDC-1 is a potential biomarker for LN and second to identify the value of serum SDC-1 as a risk factor of renal flares in comparison to traditional biomarkers such as positive anti-dsDNA or low complement components C3 and C4 by adjusting for cofounders in a multivariable approach. Nevertheless, according to the 2024 American College of Rheumatology (ACR) Guideline for the Screening, Treatment, and Management of Lupus Nephritis, “clinical judgment and patient preference are essential in deciding when to repeat kidney biopsy”, and, in our clinical setting, many patients refused repeated renal biopsy because of potential complications. These complications have been described in a wide range of patients with SLE and included bleeding (10–25%); additionally, some patients needed a blood transfusion alone without embolization or surgery, and the remaining two patients needed embolization for bleeding control, AV fistula, pain/shortness of breath, and sepsis [29,30,31]. Finally, using an rSLEDAI score ≥4 as a gold standard for LN flares, we calculated the utility values of high levels of SDC-1 and obtained a sensitivity of 66%, a specificity of 64%, a PPV of 68%, and an NPV of 62%, which are similar to those obtained for anti-dsDNA (a sensitivity of 45%, a specificity of 79%, a PPV of 71%, and an NPV of 55%). These data suggest that serum SCD-1 could be considered a complementary tool to traditional serum biomarkers used in LN (anti-dsDNA, C3, and C4). Unfortunately, as this study design was cross-sectional, we were unable to determine whether an increase in serum SDC-1 precedes the development of future relapses of LN or whether treatment modifications guided by high SDC-1 levels could prevent the worsening of the LN and its complications. Future longitudinal studies are required to investigate these important issues.

## 4. Materials and Methods

### 4.1. Study Design

This was a comparative cross-sectional study.

### 4.2. Study Population

This study recruited consecutive female patients with SLE, according to the 1997 criteria of the American College of Rheumatology (ACR) [32], from an outpatient secondary-level care center in Guadalajara, Mexico (Department of Internal Medicine–Rheumatology, Hospital General Regional No. 110, IMSS). Additional inclusion criteria were (a) female sex and (b) age > 18 years or older.

The exclusion criteria were as follows: (a) pregnant or nursing patients, (b) patients with overlapping syndromes (SLE + other autoimmune disorders), (c) patients receiving treatment with biologics, and (d) patients who had received a blood transfusion in the previous three months (these exclusion criteria were assessed based on information obtained from each patient’s chart).

*The inclusion and exclusion criteria for the controls were as follows*: (a) healthy female blood donors from the same hospital aged (b) ≥18 years without a familial relationship with SLE patients or any other connective tissue diseases.

### 4.3. Clinical Evaluation of SLE Patients and Controls

SLE patients and control participants were invited to enroll, and written informed consent was obtained from all participants prior to inclusion. The participants were interviewed using a structured chart that included sociodemographic characteristics and comorbidities. Disease activity was measured using the SLEDAI, which considers 24 clinical and laboratory findings present in the 10 days prior, corresponding to nine organ and system domains weighted according to their severity (weights of 1, 2, 4, or 8 points), with a total range of 0 to 105 points. Renal involvement was evaluated using the renal SLEDAI (rSLEDAI), derived from the renal components of the SLEDAI. For the calculation, a complete physical examination was performed (neurological, musculoskeletal, mucocutaneous, serosal, and vascular), together with laboratory tests that included a complete blood count (white blood cells and platelets), serum complement component (C3 and C4), anti-double-stranded DNA antibodies, urinalysis with urinary sediment, and quantification of proteinuria (in 24 h urine). The final SLEDAI score was obtained by summing the weights of all present descriptors.

#### 4.3.1. Assessment of Renal Disease Activity in SLE Patients

Renal involvement was evaluated using the renal SLEDAI (rSLEDAI) score [33], which is obtained by summing the scores of the renal items on the SLEDAI. The rSLEDAI includes the following items: proteinuria, pyuria, hematuria, and urinary casts. Each item is assigned either 0 points to indicate absence or 4 points to indicate presence; therefore, the maximum rSLEDAI score is 16 [33]. A higher rSLEDAI score indicates greater renal disease activity. Proteinuria greater than 0.5 g/day was used as the main indicator of renal activity or considered in conjunction with any of the following features: persistent hematuria, leucocytes in urine, or urinary casts comprising granulocytes or erythrocytes (excluding other causes). SLE patients with these features were included in the renal SLE group (LN flares). Proteinuria was considered present (4 points) when urinary protein excretion exceeded 0.5 g/24 h or when there was an increase of at least 0.5 g/24 h relative to a previously documented value. Hematuria was scored with 4 points when more than 5 red blood cells per high-power field were observed in the urinary sediment after excluding nephrolithiasis, urinary tract infection, and other non-lupus causes. Pyuria (leukocyturia) was assigned 2 points if more than 5 white blood cells per high-power field were detected in the absence of urinary tract infection or alternative etiologies. Urinary casts were scored with 4 points when hematic or granular casts were identified in the urinary sediment. The sum of these four descriptors constituted the rSLEDAI, with a maximum renal score of 16 points. According to the 2024 American College of Rheumatology (ACR) Guideline for the Screening, Treatment, and Management of Lupus Nephritis [34], LN flare is suspected in the presence of increased proteinuria, hematuria, and/or worsening kidney function. Under the renal SLEDAI definition that we used for renal flares, the worsening of kidney function is not required; instead, the quantity of proteinuria is used as a parameter. The guidelines recommend repeating kidney biopsy depending on clinical judgment and patient preference. However, in our clinical setting, repeating kidney biopsy is unusual.

#### 4.3.2. Inactive SLE Group

Patients with an SLEDAI score lower than 4 points and who did not fulfill any rSLEDAI criteria were allocated to the inactive SLE group for comparative analysis.

#### 4.3.3. Laboratory Results

All SLE patients underwent complete laboratory tests to evaluate hematological, renal, and immunological activity, such as urinalysis, including hematuria, pyuria, cellular cast, and proteinuria; complete blood cell count; platelet count; serum creatinine; creatinine clearance; proteinuria in 24 h; and immunological markers such as anti-dsDNA antibodies and C3 and C4 complement components.

#### 4.3.4. Determination of SDC-1, Anti-dsDNA Antibodies, and C3 and C4 Complement Components

A venous blood sample was obtained after 8 h of fasting. Serum was obtained via centrifugation at 3500 revolutions per minute for 15 min, and aliquots of serum were stored at −80 °C until processing. Serum SDC-1 concentrations were determined using commercially available enzyme-linked immunosorbent assay (ELISA) kits (SDC-1 (CD138) Human ELISA BioVendor, Brno, Czech Republic).

The range of determination was 8.0–256 ng/mL, with a sensitivity of 4.94 ng/mL and a coefficient of variation of 6.2%. All serum SDC-1 measurements were conducted by the same researcher, who was blinded to group allocation and clinical data. Serum antibodies against double-stranded DNA (anti-dsDNA) were measured using commercial ELISA kits (EUROIMMUN, Lübeck, Germany) according to the manufacturer’s specifications. The cutoff value for positive anti-dsDNA was 100 IU/mL. C3 and C4 serum levels were determined using commercial ELISA kits (My BioSource, San Diego, CA, USA, Cat. No. MBS700858 and MBS700030, respectively). Clinical reference for low values of C3 < 70 mg/dL and C4 < 15 mg/dL. The sensitivity of the C3 assay was 4.031 ng/mL, and that of the C4 assay was <0.41 ng/mL.

### 4.4. Statistical Analysis

Quantitative variables are expressed as means ± standard deviations (SDs), while qualitative variables are presented as frequencies and percentages (%). Differences between groups were analyzed using the independent-samples *t*-test. The chi-square test (or Fisher’s exact test, when appropriate) was used to compare proportions. Pearson’s correlation coefficient was used to assess the relationship between serum SDC-1 levels and quantitative variables.

We determined the SDC-1 level cutoff with the best sensitivity and specificity in detecting LN. We obtained the cutoff using ROC curves, which indicated a cutoff of ≥89 ng/mL. This value was used to calculate sensitivity, specificity, and positive and negative predictive values, as well as the likelihood ratio positive (LR+) and likelihood ratio negative (LR−) with their 95% confidence intervals.

To adjust for potential confounders, we performed a logistic regression analysis using the presence of LN as the dependent variable and SDC-1 ≥ 89 ng/mL, positive anti-dsDNA, and low levels of C3 or C4 as the covariates. This model was adjusted for age and disease duration. The logistic regression was run using the enter and forward LR methods. A *p*-value < 0.05 was considered significant. Odds ratios and their 95% confidence intervals were also calculated. All analyses were performed using IBM SPSS ver. 26 statistical software (Statistics/IBM Corp., Chicago, IL, USA).

### 4.5. Ethical Approval

This study was approved by the Research and Ethics Committee of the hospital in Guadalajara, Mexico (registration number R-2013-1301-40). Written informed consent was obtained from all participants prior to enrollment. This study was conducted in accordance with the Declaration of Helsinki.

## 5. Conclusions

This study found that serum SDC-1 levels were higher in flares of LN than in inactive SLE patients. SDC-1 levels were correlated with several nephritis-related variables, such as proteinuria, hematuria, rSLEDAI, and anti-dsDNA titers. Using a cutoff of ≥89 ng/mL for high serum SDC-1 levels, we found a sensitivity of 66% and a specificity of 64% for diagnosing LN, making this biomarker a useful tool for clinical decision-making in patients with SLE who are being monitored for potential renal flares. Finally, SLE patients with these levels had a 3-fold higher risk of suffering from a nephritis flare-up independent of other traditional biomarkers, such as anti-dsDNA or low C3 or C4 levels. Future studies are required to determine whether elevated SDC-1 levels are a predictor of long-term outcomes, such as chronic renal failure or poor therapeutic response, in these patients.

## Figures and Tables

**Table 1:** Characteristics of SLE patients included in this study.

Variables	SLE n = 71
Age (years), mean ± SD	43.0 ± 10.6
Female, n (%)	71 (100)
Alcohol abuse, n (%)	5 (7.0)
Smoking, n (%)	4 (5.6)
BMI (kg/m^2^), mean ± SD	27.4 ± 4.9
Disease duration (years), mean ± SD	7.9 ± 5.1
Clinical manifestations by history	
Mucocutaneous involvement, n (%)	70 (98.6)
Articular manifestations	54 (76.1)
Blood manifestations Hemolytic anemia	36 (50.7) 18 (25.4)
Leucopenia	10 (14.1)
Lymphopenia	14 (19.7)
Thrombocytopenia	6 (8.5)
Serositis	11 (8.3)
Previous renal involvement	47 (62.2)
Neurological involvement, n (%)	4 (5.6)
Clinical manifestations at the time of the study	
Mucocutaneous	19 (26.8)
Fever	4 (5.6)
Blood manifestations	2 (2.8)
Articular	4 (5.6)
Renal disease activity: renal SLEDAI score ≥4, n (%)	38 (53.5)
Proteinuria >0.5 g/24-h, n (%)	28(39.4)
Proteinuria >3.5 g/24-h, n (%)	18 (25.4)
Leukocyturia >5 white blood cells/HPF, n (%)	17 (23.9)
Hematuria >5 red blood cells/HPF, n (%)	3 (4.2)
Urinary casts, n (%)	1(1.4)
Serum creatinine, ≥1.5 (mg/dL), n (%)	5 (7.0)
rSLEDAI, mean ± SD	2.82 ± 3.05
Inactive disease, SLEDAI score = 0	33 (46.5)
Other laboratory findings	
CRP (mg/dL), mean ± SD	8.4 ± 5.4
Syndecan-1 (ng/mL), mean ± SD	108.5 ± 69.3
Anti-dsDNA (UI/mL), mean ± SD	113.1 ± 148.8
Positive anti-dsDNA ≥ 100 IU/mL, n (%)	24 (33.8)
C3, (mg/dL), mean ± SD	106.4 ± 26.1
C4, (mg/dL), mean ± SD	23.2 ± 8.8
Treatments	
Azathioprine, n (%)	37 (52.1)
Mycophenolate, n (%)	27 (38.0)
Cyclophosphamide, n (%)	7 (9.9)
Antimalarials, n (%)	27 (38.0)
Methotrexate, n (%)	10 (14.1)
Prednisone, n (%)	67 (94.4)
Prednisone < 10 mg/day	28 (39.4)
Prednisone 10–30 mg/day	32 (45.1)
Prednisone over 30 mg/day	11 (15.5)

SD: standard deviation; BMI: body mass index; rSLEDAI: renal SLEDAI; CRP: C-reactive protein; anti-dsDNA: anti-double-stranded DNA antibodies; C3 and C4: serum complement components; renal disease activity: renal SLEDAI ≥ 4.

**Table 2:** Correlations of SDC-1 and anti-dsDNA with clinical variables in SLE patients.

Variables	SDC-1 (ng/mL), n = 71		Anti-dsDNA (UI/mL), n = 71	
	r	*p*	r	*p*
Age	−0.17	0.15	−0.33	0.006 **
BMI (kg/m^2^), mean ± SD	−0.14	0.007 **	−0.20	0.16
Hemoglobin (g/dL), mean ± SD	0.06	0.64	0.24	0.04
Leukocytes (1 × 10^3^/μL), mean ± SD	−0.03	0.80	0.07	0.58
Lymphocytes (1 × 10^3^/μL), mean ± SD	−0.18	0.31	0.02	0.92
Platelets (1 × 10^3^/μL), mean ± SD	−0.30	0.01 **	−0.02	0.90
Serum creatinine, (mg/dL), mean ± SD	0.15	0.22	−0.05	0.70
Serum glucose (mg/dL), mean ± SD	−0.03	0.84	−0.24	0.08
Total cholesterol (mg/dL), mean ± SD	−0.29	0.05 *	−0.09	0.56
Triglycerides (mg/dL), mean ± SD	0.11	0.94	0.24	0.10
HDL cholesterol (mg/dL), mean ± SD	−0.04	0.80	−0.16	0.28
Disease duration	−0.16	0.18	0.04	0.76
SLEDAI				
Hematuria (1 × 10^3^/μL), mean ± SD	0.30	0.01 *	−0.03	0.82
Leukocyturia (1 × 10^3^/μL), mean ± SD	0.20	0.10	−0.01	0.91
Proteins in urine (g/24-h)	0.33	0.005 **	0.39	0.001 ***
Creatinine clearance	−0.01	0.96	0.14	0.24
rSLEDAI	0.32	0.006 **	0.12	0.31
Prednisone doses (mg/day)	0.37	0.002 **	0.19	0.11
SDC-1 (ng/mL)	–	–	0.33	0.006 **
Anti-dsDNA (UI/mL)	0.33	0.006 **	–	–
C3 complement component (mg/dL)	−0.16	0.19	−0.10	0.40
C4 complement component (mg/dL)	0.05	0.67	−0.15	0.21
CRP (mg/dL)	−0.12	0.40	0.01	0.93

BMI: body mass index; HDL: high-density lipoprotein; UI: international units; rSLEDAI: renal SLEDAI; anti-dsDNA: antibodies against double-stranded DNA. Pearson correlation test, * *p* < 0.05, ** *p* < 0.01, *** *p* < 0.001. *p*-values with significance are shown in bold.

**Table 3:** Utility values of SDC-1 ≥ 89 ng/mL and anti-dsDNA ≥ 100 UI/mL as biomarkers for lupus nephritis in SLE patients.

Utility Values of the Assay	Lupus Nephritis	
	SDC-1 ≥ 89 ng/mL	Anti-dsDNA ≥ 100 UI/mL
Sensitivity % (95% CI)	66 (49–79)	45 (29–60)
Specificity % (95% CI)	64 (46–79)	79 (62–91)
PPV % (95% CI)	68 (51–81)	71 (49–86)
NPV % (95% CI)	62 (44–77)	55 (40–71)
LR+ % (95% CI)	1.81 (1.09–3.00)	2.11 (1.0–4.45)
LR− % (95% CI)	0.54 (0.32–0.90)	0.70 (0.52–0.94)

Prevalence % (95% CI) 54 (42–65); PPV: positive predictive value; NPV: negative predictive value; LR+: positive likelihood ratio; LR−: negative likelihood ratio.

**Table 4:** Comparison of clinical variables between lupus nephritis and inactive SLE patients.

Variables	Overall SLE n = 71	Lupus Nephritis n = 38	Inactive SLE n = 33	*p*
Age (years), mean ± SD	43.0 ± 10.6	42.1 ± 9.9	44.1 ± 11.4	0.442
Disease duration (years), mean ± SD	7.9 ± 5.1	7.6 ± 4.7	8.2 ± 5.5	0.591
BMI	27.6 ± 4.9	26.9 ± 4.8	28.2 ± 5.0	0.337
SLEDAI mean ± SD	4.5 ± 3.9	7.0 ± 3.0	0.5 ± 0.8	<0.001 **
Glucocorticoid use, n (%)	71 (100)	38 (100)	33 (100)	–
- GC dose > 10 mg/day, n (%)	43 (60.6)	28 (73.7)	15 (45.5)	0.016 *
Immunosuppressive drugs, n (%)	55 (77.5)	32 (84.2)	23 (69.7)	0.166
- Azathioprine, n (%)	37 (52.1)	22 (57.9)	15 (45.5)	0.302
- Cyclophosphamide, n (%)	7 (9.9)	4 (10.5)	3 (9.7)	0.880
- Mycophenolate, n (%)	27 (38.0)	16 (42.1)	11 (34.4)	0.459
- Methotrexate, n (%)	10 (14.1)	5 (13.2)	5 (15.2)	0.813
CRP (mg/dL), mean ± SD	8.4 ± 5.4	8.1 ± 4.4	8.6 ± 6.3	0.720
C3 complement component (mg/dL), mean ± SD	106.4 ± 26.1	103.0 ± 23.5	110.3 ± 28.6	0.238
C4 complement component (mg/dL), mean ± SD	23.2 ± 8.8	21.0 ± 6.8	25.9 ± 10.2	0.018 *
Positive anti-dsDNA ≥ 100 UI/mL, n (%)	24 (33.8)	17 (44.7)	7 (21.2)	0.037 *
SDC-1 (ng/mL), mean ± SD	108.5 ± 69.3	127.4 ± 76.2	86.7 ± 53.5	0.011 *

SLE: systemic lupus erythematosus; SD: standard deviation; lupus nephritis: rSLEDAI ≥ 4; inactive SLE: SLEDAI <4 points and no rSLEDAI criteria fulfilled. Comparisons between proportions were conducted with chi-square or Fisher’s exact test (when required). Comparisons between means were evaluated with Student’s *t*-test for independent samples. * *p* < 0.05, ** *p* < 0.001. *p*-values with significance are shown in bold.

**Table 5:** Logistic regression analysis evaluating factors associated with lupus nephritis.

Predictor Criterion	Enter Method			Forward Method		
Variable	OR	CI (95%)	*p*	OR	CI (95%)	*p*
Age, years	1.00	0.95–1.06	0.98		Not significant	
Disease duration, years	1.00	0.90–1.12	0.92		Not significant	
SDC-1, ≥89 ng/mL	2.91	1.04–8.17	0.04	3.37	1.27–8.93	0.02
Positive anti-dsDNA	2.66	0.81–8.71	0.11		Not significant	
Low C3 or C4 levels	1.38	0.23–8.13	0.73		Not significant	

## Data Availability

The data presented in this study are available on request from the corresponding author due to privacy restrictions.
